# A rare case of CNS hemangiopericytoma presenting with papilledema

**DOI:** 10.3205/oc000121

**Published:** 2019-08-20

**Authors:** Sanjana B. Singh, Narayanaswamy Vanathi, Virna M. Shah

**Affiliations:** 1 Department of Neuro Ophthalmology, Aravind Eye Hospital & Postgraduate Institute of Ophthalmology, Coimbatore, India

**Keywords:** hemangiopericytoma, central nervous system, papilledema

## Abstract

Hemangiopericytoma rarely affects the central nervous system (CNS) and usually presents to neurologists with neurological symptoms. We report a rare case of large CNS hemangiopericytoma which presented to an ophthalmologist with only signs of mild defective vision and papilledema.

## Introduction

Hemangiopericytoma is a rare aggressive type of soft tissue sarcoma which arises from pericytes of the capillaries [1] mostly involving the musculoskeleton system. Presentation in the central nervous system (CNS) is very rare, accounting for less than 1% of all intracranial tumors and 2–4% of meningeal tumours, and can mimic a meningioma [2]. We report a case of a large intracranial hemangiopericytoma in the left temporal region that presented to us with blurring of vision, headache, and papilledema. There were no sensory or motor disturbances. This is one of the few cases of rare intracranial hemangiopericytoma presenting to an ophthalmologist with only ocular symptoms and no systemic signs.

## Case description

**Figure 1:**
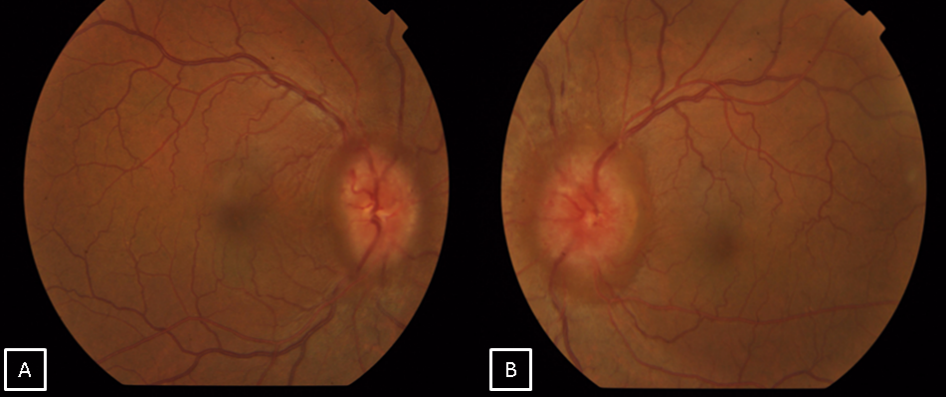
Fundus pictures of the right (A) and left eye (B) showing bilateral optic disc edema suggestive of papilledema

**Figure 2:**
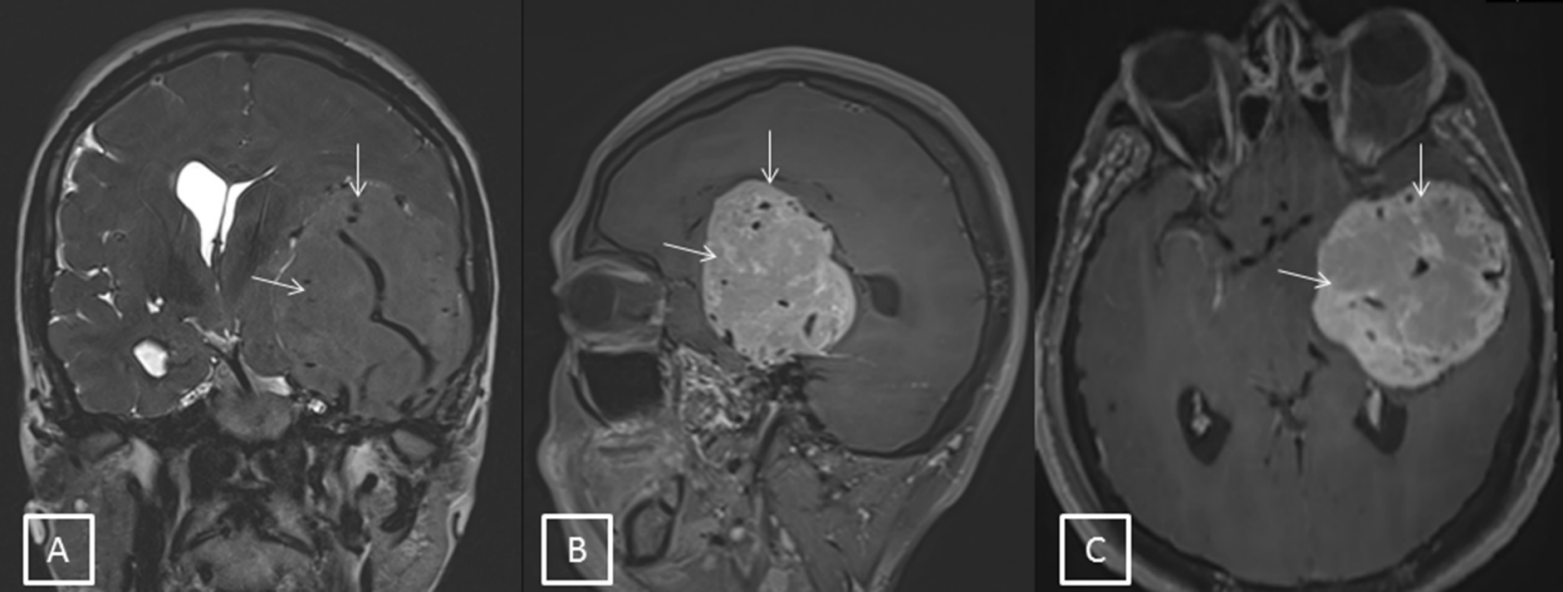
A) Coronal T2-weighted MRI brain showing an iso-hyperintense large mass lesion in the tempo-parietal lobe (white arrows). B,C): Post contrast sagittal and axial cuts showing an intense heterogeneous enhancement of the lesion (white arrows).

A forty-one-year old male presented with defective vision in the right eye since 2 months. He also gave a history of transient obscuration of vision in the right eye with associated headache. There was no history of associated vomiting, loss of consciousness, weakness in any part of the body, diplopia, or seizures. On examination, his best corrective visual acuity was 20/30 in the right eye and 20/20 in the left eye. Anterior segment examination of both eyes was within normal limits. Fundus examination of both eyes showed disc edema suggestive of papilledema (Figure 1 (Fig. 1)). Colour vision and extraocular movements were normal in both eyes. Humphrey visual field analyzer showed an enlargement of the blind spot in both eyes. Higher functions and other cranial nerves examinations were within normal limits. The patient was diagnosed to have bilateral papilledema and was advised magnetic resonance imaging (MRI) of the brain and orbit. MRI showed a large mass lesion measuring about 7x6x6 cm in maximum dimension over the left petrous temporal bone with prominent vascular channels within the lesion suggestive of hemangiopericytoma (Figure 2 (Fig. 2)). Mass effect in the form of midline shift to the right was noted with mild dilatation of the right ventricle suggestive of intracranial hypertension. Spectroscopy revealed elevated myoinositol and choline peaks. Limited computerized tomography showed erosion of the anterior aspect of the petrous portion of the temporal bone on the left side. The patient was referred to a neurosurgeon for further management. The patient decided against treatment and deceased within two months.

## Discussion

Hemangiopericytoma is encountered in adults in their fifth decade of life with a slight male predilection [3]. Based on histology, hemangiopericytoma of the CNS are of two types, namely differentiated and anaplastic, of which the latter is more aggressive. The differentiated type of intracranial hemangiopericytoma is considered a World Health Organization grade II neoplasm, whereas the anaplastic variant is classified as grade III neoplasm [4]. Hemangiopericytoma mimics meningioma, but has a much more agressive course [2]. One third of the cases exhibit malignant features like metastasis or local invasion and recurrence. Bone, liver, CNS, and abdominal cavity are the most common sites of metastasis [5]. However, the prognosis depends upon location and size of the tumor as well as the presence of metastasis. Tentorial and posterior fossa tumors are found to be more aggressive than supratentorial tumors. Large tumours (≥6 cm) and non-skull base lesions have an increased risk of early recurrence after treatment. A treatment modality of intracranial hemangiopericytoma is total surgical excision with pre-operative catheter embolisation and adjuvant radiotherapy to reduce the incidence of recurrence [5].

Our case presented to us with defective vision and headache of 2 months duration without any motor or sensory disturbances, though the size of the tumor was large on MRI. We report this case to lay emphasis on the fact that intracranial hemangiopericytoma can present to an ophthalmologist which requires timely referral.

## Notes

### Competing interests

The authors declare that they have no competing interests.
